# Impact of land-use on malaria transmission in the Plateau region, southeastern Benin

**DOI:** 10.1186/1756-3305-6-352

**Published:** 2013-12-12

**Authors:** Arthur Sovi, Renaud Govoétchan, Filémon Tokponnon, Hermine Hounkonnou, Rock Aïkpon, Fiacre Agossa, Virgile Gnanguenon, Albert S Salako, Christian Agossou, Razaki Ossè, Mariam Okè, Dina Gbénou, Achille Massougbodji, Martin Akogbéto

**Affiliations:** 1 Centre de Recherche Entomologique de Cotonou, Cotonou 06 BP 2604, Benin; 2 Faculté des Sciences et Techniques de l’Université d’Abomey Calavi, Cotonou 01 BP 4521, Bénin; 3 Programme Nationale de Lutte contre le Paludisme, Cotonou, Benin; 4 World Health Organisation, Cotonou, Benin; 5 Faculté des Sciences de la Santé de l’Université d’Abomey Calavi, Cotonou, Benin; 6 School of Public Health, University of Colorado, Denver, USA

**Keywords:** *An. gambiae*, Transmission, Resistance, Fishpond basins, Market-garden

## Abstract

**Background:**

The goal of the study is to investigate if local agricultural practices have an impact on malaria transmission in four villages located in the same geographical area within a radius of 15 kilometers. Among the villages, one (Itassoumba) is characterized by the presence of a large market garden and fishpond basins, the three others (Itakpako, Djohounkollé and Ko-koumolou) are characterized by traditional food-producing agriculture.

**Methods:**

Malaria transmission was evaluated using human-landing catches, both indoors and outdoors, two nights per month for 12 months. Field collected females *An. gambiae s.l.* were exposed for 1 hour to 0.75% permethrin and 0.05% deltamethrin using WHO insecticide susceptibility test kits and procedures. The presence of the *kdr* mutation was analyzed by PCR.

**Results:**

*Anopheles gambiae s.s* form M (93.65%), was identified as the main malaria vector. Its susceptibility level to pyrethroids was the same (p > 0.05) in all villages. *kdr* mutation frequencies are 81.08 in Itakpako, 85 in Itassoumba, 79.73 in Djohounkollé and 86.84 in Ko-Koumolou (p = 0.63). The entomological inoculation rate ranged from 9.62 to 21.65 infected bites of *An. gambiae* per human per year in Djohounkollé, Itakpako and Ko-Koumolou against 1159.62 in Itassoumba (p < 0.0001).

**Conclusion:**

The level of resistance of *An. gambiae* to pyrethroids was the same in the four villages. The heterogeneous character of malaria epidemiology was confirmed. The creation of fishponds basins and the development of market-gardening activities increased drastically the malaria transmission in Itassoumba.

## Background

In Africa, malaria transmission varies according to ecological features. The relations which exist between the host, the parasite and the vector vary according to environmental conditions. This creates various epidemiological features. Carnevale *et al.*[1], Mouchet *et al.*[2], Fontenille *et al*. [3] described three areas of malaria transmission in Africa: the area with stable, intense and permanent malaria transmission, the area with unstable malaria and episodic transmission and the area with intermediate stability with seasonal recrudescence transmission. But within these areas, the transmission is not univocal. Heterogeneous biotopes are often announced [4]. The malaria transmission is thus an environmental problem. Different features in the transmission are recognized today: urban malaria, coastal lagoon malaria, malaria of the rice cultivation areas, malaria in altitude area, malaria in dams and forest areas [4-7].

In the southern Sahara countries, the agricultural policy is more and more oriented towards the creation of small dams in order to ensure the food safety of the populations. The increase in population in sub-Saharan Africa and the challenges in agriculture for several decades leads to the development of hydraulic installation, particularly irrigated rice growing areas, market gardening [5] and fish breeding. However, if agricultural dams increase the production of cereals and market-gardening products, they also have a major effect on the endemicity of malaria, because of the creation of mosquito breeding sites suitable for the development of the earlier ecological stages of malaria vectors [8]. Moreover, the works of Manga et *al*. [9] in Cameroun, Klinkenberg et *al*. [10] in Ghana and Yadouléton et *al*. [11] in Benin showed that market-gardening sites are also an area suitable for the development of mosquito larvae. This is explained by the pockets of water constantly maintained by the gardeners for watering their plants that form permanent breeding sites for *Anopheles* larvae. Moreover, Yadouléton *et al.*[11] showed that malaria transmission is higher in habitation close to market-gardening sites compared to those which are distant. The rice fields, for example, generally cause the mosquitoes proliferation, and constitute an environmental modification model of which entomological impact is variable depending on the local situation and the malaria stability [12]. In addition, several authors have reported that, the pyrethroids use in agricultural areas, especially, in the market-gardening areas is a known practice [13-15]. However, according to Akogbéto *et al.*[16], several mosquito species, in particular *An. gambiae*, lays their eggs in breeding sites located in the cultivated areas and are likely to be exposed to pyrethroids during the treatments against agricultural pests. According to these same authors, residues of pyrethroids could be found in the soil and could exert a selection pressure on mosquito populations during their development cycle. It is therefore possible that the different agricultural practices implemented have an impact on the malaria vectors resistance status as well on the transmission of the parasites responsible for this disease.

The current study aims to investigate malaria transmission in four villages (Itassoumba, Ko-Koumolou, Djohounkollé and Itakpako) located within a radius of 15 kilometers in the rural area of Benin. Among the four villages, Itassoumba is characterized by a particular land use with the presence of fish breeding sites with a large market garden, contrary to the other three villages where only the culture of cultivating cereals and food stuffs is practiced. In 2011 and 2012, mosquitoes catches were carried out in the four villages. The entomological indicators recorded in Itassoumba were compared to those of the other three villages.

## Methods

### Study area

**Figure 1:**
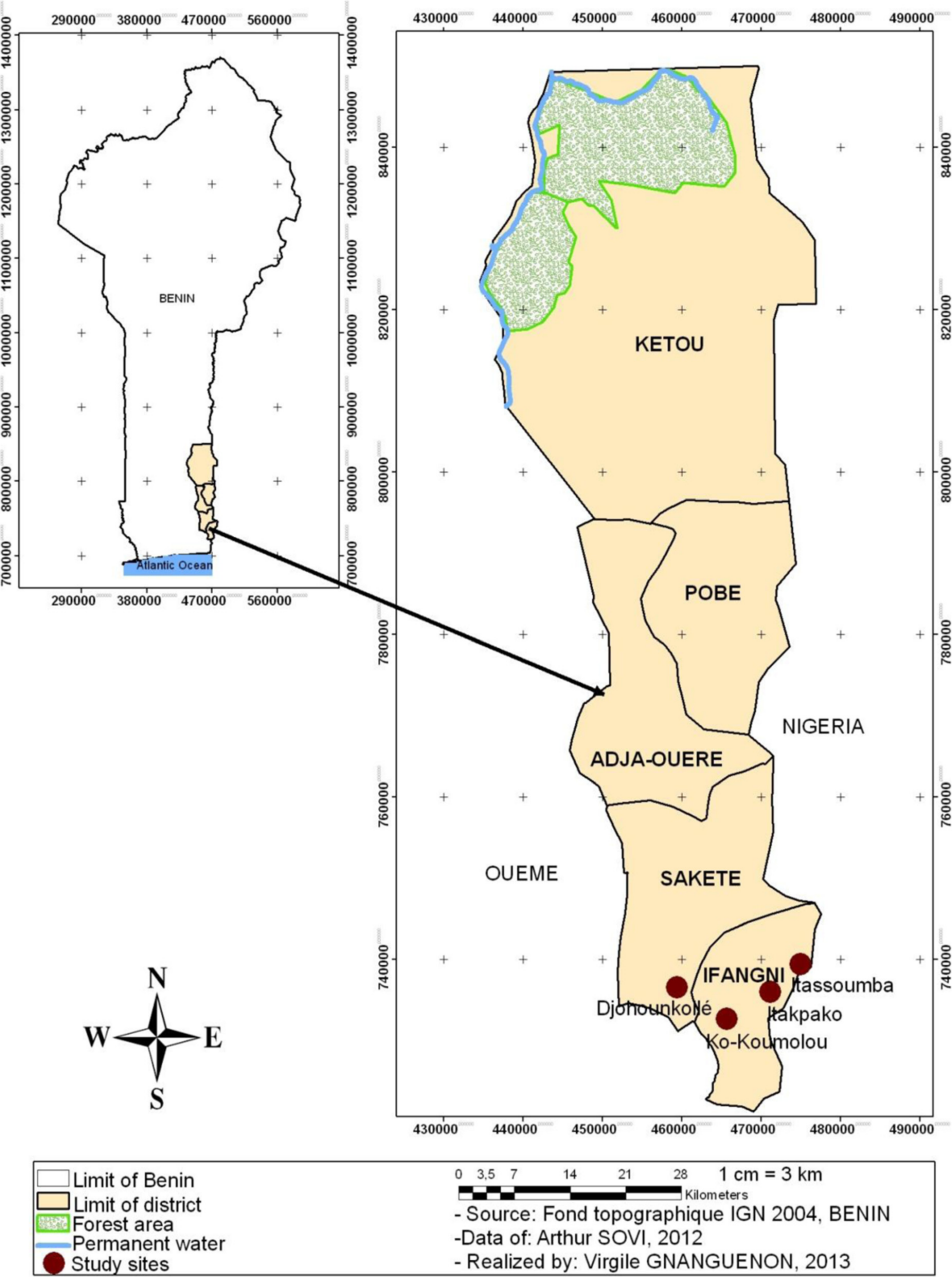
Map showing the study villages in the region of Plateau (Southeastern Benin).

This study was carried out from July 2011 to June 2012 (12 months) in the Plateau’s region, located in Southeastern Benin, West Africa, and more precisely in four villages of Ifangni and Sakété districts. Itakpako, Itassoumba and Ko-Koumolou are in Ifangni district, and Djohounkollé in Sakété district (Figure 1). This region has a little uneven relief with the presence of some depressions. The climate is guinean with two dry seasons and two rainy seasons which alternate during the year: a long rainy season from March to July, a short dry season during August, then a short rainy season from September to November and finally, a long dry season from December to February. The Plateau region holds a lot of swamps used for market gardening and the cultivation of different species of nursery trees. At the country scale, malaria is endemic and stable with seasonal upsurges in the Plateau region. The number of malaria cases recorded there in 2011 and 2012 were 40512 and 33745 respectively [17,18]. At the national level 7.4% and 6% of all malaria cases were recorded in this region in 2011 and 2012. In 2011, 43 deaths were recorded in the Plateau region against 60 deaths in 2012 [17,18]. A cross sectional investigation carried out on children under five years in June 2012 showed that malaria prevalence was 57.5%, 15% and 15% respectively at Itassoumba, Ko-Koumolou, Djohounkollé (Tokponnon, unpublished observation).

**Figure 2:**
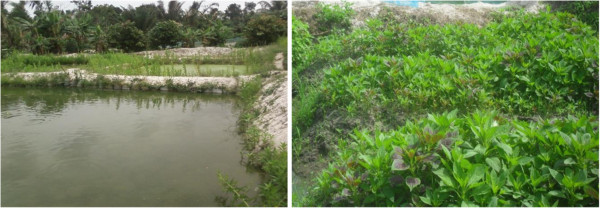
Fishponds basins and market garden site of Itassoumba.

In 2011, a large scale distribution of Long Lasting Insecticidal Nets (LLINs) was carried out in this region so that the individual coverage rate of mosquito nets in 2012 was 91.39% in Itassoumba, 98.78% in Itakpako, 106.73% in Ko-Koumolou and 93.78% in Djohounkollé (Sovi, unpublished observation). Itassoumba is a village located near a large market-gardening site where there are several fishponds (Figure 2) contrary to Itakpako, Ko-Koumolou and Djohounkollé where only the culture of cultivating cereals and food stuffs is practiced. The distance between the breeding sites and households varies generally between 0.5 and 2.1 km in the four villages.

The market-gardening and fishponds field of Itassoumba covers approximately an area of 4 hectares. In this perimeter, we found 128 basins where breeding of mainly tilapia and to a lesser extent catfishes nourished with various types of provender (Coppens, Legouessant, Multifeed or Aquafeed) used according to the development stage of alevins or fish. The two types of fishes are bred in different basins and the provender is often thrown on the surface of the water to feed them. One fish breeding site is generally maintained by one person. As time goes on, new basins are dug in order to increase fish production on the site because of the unceasingly increasing requests from the market. The breeding of fishes is done throughout the year and the sale of these fishes is done at the markets in the communities of Ifangni and the close localities of Nigeria. The production of vegetables is seasonal in the market gardens of Itassoumba from December to February corresponding to the period of the dry season where vegetables are generally not available on the market. Vegetables are produced on plots and watering is done with water from fishponds.

### Sampling of mosquito populations

#### ***Human landing catch***

To measure the malaria transmission in each village, we collected *Anopheles*. This gathering method of *Anopheles* enabled us to evaluate the biting rate and the frequency of infected biting for each village. In each village, for one capture session, 4 human volunteers were used as bait for both indoor and outdoor mosquito collections in 2 houses, according to the human landing catch technique. Two successive capture sessions were carried out per month during 10 months in each village totaling 8 man night catches per month per village, then 80 man night catches per 10 months per village. Another collections of mosquitoes were carried out on 2 nights (1 in December 2011 and 1 in January 2012) totaling 8 man night catches per 2 months per village. These catches were then conducted for a period of 12 months totaling 88 man night catches per year per village. Mosquito collections were made from 9 pm to 05 am the following morning.

The captured mosquitoes were identified the next morning using the morphological identification key of Gillies & de Meillon [19]. The head and thorax of each *Anopheles* is preserved on silicagel for infectivity determination using circumsporozoite protein (CSP) ELISA technique for *P. falciparum* detection. From mosquitoes collected and the ELISA test results, we determined the human biting rate, the sporozoitic index and the entomological inoculation rate (EIR) for each village.

#### ***Collection of An. gambiae larvae***

Larvae and pupae of *An. gambiae* were collected by the method of “dipping”. These larvae and pupae were kept separately in labeled bottles and were reared in the insectarium of Centre de Recherche Entomologique de Cotonou (CREC) until they emerged into adults mosquitoes. Females aged from 2 to 5 days were used for WHO susceptibility bioassay under laboratory conditions (25°C ± 2°C and 80% ± 4% relative humidity).

### Susceptibility of *An. gambiae* to insecticides

WHO susceptibility bioassays were performed using unfed females of *An. gambiae s.l*, aged from 2 to 5 days. These bioassays were carried out with impregnated papers of deltamethrin (0.05%) and permethrin (0.75%). 25 females were introduced into treatment tube for 60 minutes. The number of knocked down mosquitoes were recorded every ten minutes during the period of exposure. Field collected mosquitoes exposed to non-impregnated papers were used as control. After the 60 minutes of exposure, the mosquitoes are transferred into observation tubes and were fed with 10% honey solution then maintained under observation for 24 hours. At the end of the observation period, mortality rate was calculated. According to WHO [20], a mortality rate higher than 97% means that the population of mosquito tested is susceptible, a mortality rate between 90 and 97% means a suspicion of resistance and a mortality rate lower than 90% means that the mosquito population tested is resistant. After the tests, the dead and alive mosquitoes are preserved separately on silicagel and are stored at -20°C for molecular characterization by PCR.

### Infection and molecular characterization of the populations of *An. gambiae* by PCR: species, molecular forms and *kdr* Leu-phe mutation

The head and thorax of each *Anopheles* captured and preserved on silicagel were used for the detection of infection by CSP ELISA method [21,22]. Approximately 30 to 43 females of *An. gambiae* of each village resulting from the susceptibility tests performed with deltamethrin were analyzed by PCR. The species of *An. gambiae s.l.* complex and the molecular forms of *An. gambiae s.s* were identified according to the protocols described by Scott *et al.*[23] and Favia *et al.*[24]. The *kdr* mutation was characterized according to the protocol of Martinez-Torrez *et al.*[25].

### Statistical analysis

The biting rate was calculated as the ratio of the number of mosquitoes captured over the number of collectors per night. The infection rate of mosquitoes was evaluated by dividing the number of mosquitoes that were positive for CSP ELISA test over the total number of mosquitoes tested. The EIR is defined as being the number of infective bites received by human per unit time and was evaluated using the product of the biting rate and the sporozoitic index.

To compare the variability of *An. gambiae* human biting rate (HBR) and the EIR between the villages, we used the balanced regression of Poisson followed by an analysis of the deviance using the ratio test of probability. For the variability of the infectivity rate and frequencies of *kdr* between the villages, we used the comparison of the proportions test of Chi^2^. All these analyses were carried out with the Software R 2.14.1 [26].

### Ethical clearance

This study was approved by the National Ethic Committee of Health Research of Benin, the Ministry of Health and the Centre de Recherche Entomologique de Cotonou (CREC). The volunteer mosquito collectors gave their consent before participating in the study. They were vaccinated against yellow fever and treated each time against malaria based on the Rapid Diagnostic Test of *P. falciparum.*

## Results

### Diversity of culicidae

A total of 5230 mosquitoes belonging to 13 species were collected during the 12 months of the study. The distribution of the various species per village is presented in Table 1.

**Table 1:** Diversity of mosquitoes in the four villages from July 2011 to June 2012

	**Villages**					
**Species**	**Itakpako**	**Itassoumba**	**Djohounkollé**	**Ko-Koumolou**	**Total/genus**	**Genus (%)**
*Anopheles gambiae*	43	3994	58	18	4130	78.97
*Anopheles pharoensis*	6	2	2	4		
*Anopheles ziemanni*	0	2	0	0		
*Anopheles coustani*	0	1	0	0		
*Aedes aegypti*	3	9	8	7	674	12.89
*Aedes vittatus*	0	0	607	4		
*Aedes palpalis*	8	9	13	6		
*Culex quinquefasciatus*	31	26	97	21	305	5.83
*Culex decens*	3	4	4	6		
*Culex nebulosus*	17	54	16	18		
*Culex tigripes*	0	0	2	1		
*Culex annulioris*	0	0	5	0		
*Mansonia africana*	14	75	16	16	121	2.31
TOTAL	125	4176	828	101	5230	100

Out of the total mosquitoes caught, 78.97% (4130/5230) were *Anopheles* genus, 12.89% (674/5230) of *Aedes*, 5.83% (305/5230) of *Culex* and 2.32% (121/5230) of *Mansonia* (Table 1). These four mosquito genera were present in each of the four villages but in variable proportions. The greatest specific diversity was observed within the genus *Culex* with five species (*Cx. quinquefasciatus*, *Cx. nebulosus*, *Cx. descens*, *Cx. annulioris* and *Cx. tigripes*), followed by the genus *Anopheles* with four species (*An. gambiae, An. ziemanni, An. pharoensis* and *An. coustani*).

*An. gambiae* was the most abundant species in all the four villages. It accounted for 78.64% (4113/5230) of the total collected mosquitoes.

For the same study period, 97.11% (3994/4113) of *An. gambiae* mosquitoes collected were captured in the market-gardening and fishponds village of Itassoumba against only 1.41% (58/4113) in Djohounkollé, 1.04% (43/4113) in Itakpako, and 0.44% (18/4113) in Ko-Koumolou. In Djohounkollé, 73.31% (607/828) of the mosquitoes caught were *Aedes vittatus*.

In addition to the *Anopheles* abundance in Itassoumba, *Mansonia africana* was also present and accounts for 1.80% (75/4176) of sampled mosquitoes. The presence of this species is probably due to the presence of swamps in the village. Globally, the land use in Itassoumba affected *Anopheline* densities (n = 3999) but not *Culicine* densities (n = 177).

### Infectivity of *An. gambiae*

Table 2 shows the number of mosquitoes analyzed by CSP ELISA and the number of mosquitoes found positive each month. The monthly variation of the infection rate of the mosquitoes during the study period in each village is indicated in the same table. Given the weak number of *An. gambiae* mosquitoes collected during certain months in the majority of the villages, we cumulated our samples over the entire study period to get a higher number of mosquitoes to test. The mean annual sporozoitic index has been 0.06 in Itakpako (2 head-thoraxes positive over 33 tested); 0.07 in Itassoumba (111 positive head-thoraxes over 1519 tested); 0.04 (2 positive head-thoraxes over 52 tested) in Djohounkollé and 0.29 (positive head-thoraxes over 17 tested) in Ko-Koumolou (Table 2). The index is similar in the villages of Itakpako, Itassoumba and Djohounkollé (p = 0.34). A significant difference was obtained between the sporozoitic index recorded in Ko-Koumolou and those obtained in Itakpako and Itassoumba (p < 0.05). These results show that, the infection rate of *An. gambiae* is the same in the villages except in Ko-Koumolou where it is higher, but the ELISA test was performed on only 17 mosquitoes.

**Table 2:** Monthly variation of sporozoitic index from July 2011 to July 2012

**Villages**		**July-11**	**Aug-11**	**Sept-11**	**Oct-11**	**Nov-11**	**Dec-11**	**Jan-12**	**Feb-12**	**Mar-12**	**Apr-12**	**May-12**	**June-12**	**Total (1 year)**	**CI (95%)**
	Thorax	5	1	0	1	2	0	0	0	0	0	3	21	33	
Itakpako	Thorax^+^	0	0	0	1	0	0	0	0	0	0	0	1	2	
	S	0	0	-	1	0	-	-	-	-	-	-	0.05	**0.06**^**a**^	[0.01-0.20]
	Thorax	47	25	22	78	112	41	222	290	202	160	150	170	1519	
Itassoumba	Thorax^+^	0	11	2	12	10	3	12	15	24	6	8	8	111	
	S	0	0.44	0.09	0.15	0.09	0.07	0.05	0.05	0.12	0.04	0.05	0.05	**0.07**^**a**^	[0.06-0.09]
	Thorax	2	1	0	4	3	0	0	0	0	1	12	29	52	
Djohounkollé	Thorax^+^	0	0	0	0	0	0	0	0	0	0	0	2	2	
	S	0	0	-	0	0	-	-	-	-	0	0	0.07	**0.04**^**ac**^	[0.00-0.13]
	Thorax	7	1	1	1	4	0	0	0	0	1	1	1	17	
Ko-Koumolou	Thorax^+^	3	0	0	0	1	0	0	0	0	0	0	1	5	
	S	0.43	0	0	0	0.25	-	-	-	-	0	0	1	**0.29**^**c**^	[0.10-0.56]

S: sporozotic index; CI: confidence interval; ^a, c, ac^Values with the same superscript do not differ significantly (p > 0.05), ^+^: positive.

### Biting and entomological inoculation rates

During the twelve months covered by the study, *An. gambiae* was particularly aggressive in Itassoumba compared to the three other villages (p < 0.05). In Itassoumba, a man received an average of 16566.02 bites per year against only 178.35 in Itakpako, 240.56 in Djohounkollé and 74.66 in Ko-Koumolou for the same period (Table 3). A significant difference is observed between the biting rates obtained in the four villages (P < 0.05). As a result, the ratio is one bite of *An. gambiae* per man per year in each of the three villages (Itakpako, Djohounkollé and Ko-Koumolou) located far from the market-gardening and fishponds area against 100 bites per man per year in Itassoumba.

**Table 3:** Monthly variation of human biting rate and entomological inoculation rate in four villages from July 2011 to June 2012

**Villages**	**Parameters**	**July-11**	**Aug-11**	**Sept-11**	**Oct-11**	**Nov-11**	**Dec-11**	**Jan-12**	**Féb-12**	**Mar-12**	**Apr-12**	**May-12**	**June-12**	**Total (1 year)**
**Itakpako**	Total Vect	7	5	1	1	3	0	0	0	0	0	3	23	43
	Man night	8	8	8	8	8	4	4	8	8	8	8	8	88
	HBR	0.88	0.63	0.13	0.13	0.38	0	0	0	0	0	0.38	2.88	**178.35**^**a**^
	S	0	0	-	1	0	-	-	-	-	-	-	0,05	0,06
	EIR	0	0	-	0,13	0	-	-	-	-	-	-	0,14	**10,70**^**a**^
	**CI**	[0–0.06]	[0–0.31]	-	[0–0.31]	[0–0.15]	-	-	-	-	-	-	[0.09-0.20]	[7.3-14.6]
**Itassoumba**	Total Vect	298	239	134	171	477	136	223	524	319	500	510	463	3994
	Man night	8	8	8	8	8	4	4	8	8	8	8	8	88
	HBR	37.25	29.88	16.75	21.38	59.62	34	55.75	65.50	39.88	62.50	63.75	57.88	**16566.02**^**b**^
	S	0	0.44	0.09	0.15	0.09	0.07	0.05	0.05	0.12	0.04	0.05	0.05	0.07
	EIR	0	13.14	1.51	3.21	5.36	2.38	2.79	3.27	4.79	2.50	3.19	2.89	**1159.62**^**b**^
	**CI**	[0–0.01]	[12.65-13.65]	[1.35-1.71]	[3.15-3.43]	[5.17-5.48]	[2.26-2.74]	[2.70-03.13]	[3.19-3.46]	[4.63-4.84]	[2.26-2.43]	[2.99-3.51]	[2.64-2.94]	[1157.15-1160.45]
**Djohounkollé**	Total Vect	3	3	0	6	4	0	0	0	0	1	12	29	58
	Man night	8	8	8	8	8	4	4	8	8	8	8	8	88
	HBR	0.38	0.38	0	0.75	0.50	0	0	0	0	0.13	1.5	3.63	**240.56**^**c**^
	S	0	0	-	0	0	-	-	-	-	0	0	0.07	0.04
	EIR	0	0	-	0	0	-	-	-	-	0	0	0.25	**9.62**^**c**^
	**CI**	[0–0.15]	[0–0.31]	-	[0–0.08]	[0–0.10]	-	-	-	-	[0–0.31]	[0–0.03]	[0.19-0.32]	[07.30-12,41]
**Ko-Koumolou**	Total Vect	7	1	1	1	4	0	0	0	0	1	1	2	18
	Man night	8	8	8	8	8	4	4	8	8	8	8	8	88
	HBR	0.88	0.13	0.13	0.13	0.50	0	0	0	0	0.13	0.13	0.25	**74.66**^**d**^
	S	0.43	0	0	0	0.25	-	-	-	-	0	0	1	0,29
	EIR	0.38	0	0	0	0.13	-	-	-	-	0	0	0,25	**21.65**^**d**^
	**CI**	[0.24-0.56]	[0–0.31]	[0–0.31]	[0–0.31]	[0.04-0.30]	-	-	-	-	[0–0.31]	[0–0.31]	[0.05-0.80]	[18.25-25.55]

Total Vect: total of vectors; HBR: human biting rate; EIR: entomological inoculation rate; ^a, b, c, d^The same parameters with different superscript are statistically different (p < 0.05).

Various EIR of *An. gambiae* were recorded each month in the four villages. Except in July 2011, the EIR was very high in Itassoumba compared to the other three villages (p < 0.05). In July 2011, no mosquito was found infected in Itassoumba so a null EIR was recorded. After the 12 months covered by the study, The EIR varied from one village to the other. In Itassoumba, a man received an average of 1159.62 infective bites of *An. gambiae* against only 9.62 in Djohounkollé, 10.70 in Itakpako and 21.65 in Ko-Koumolou for the same period (p < 0.0001) (Table 3).

### Susceptibility of *An. gambiae* to deltamethrin and permethrin

**Figure 3:**
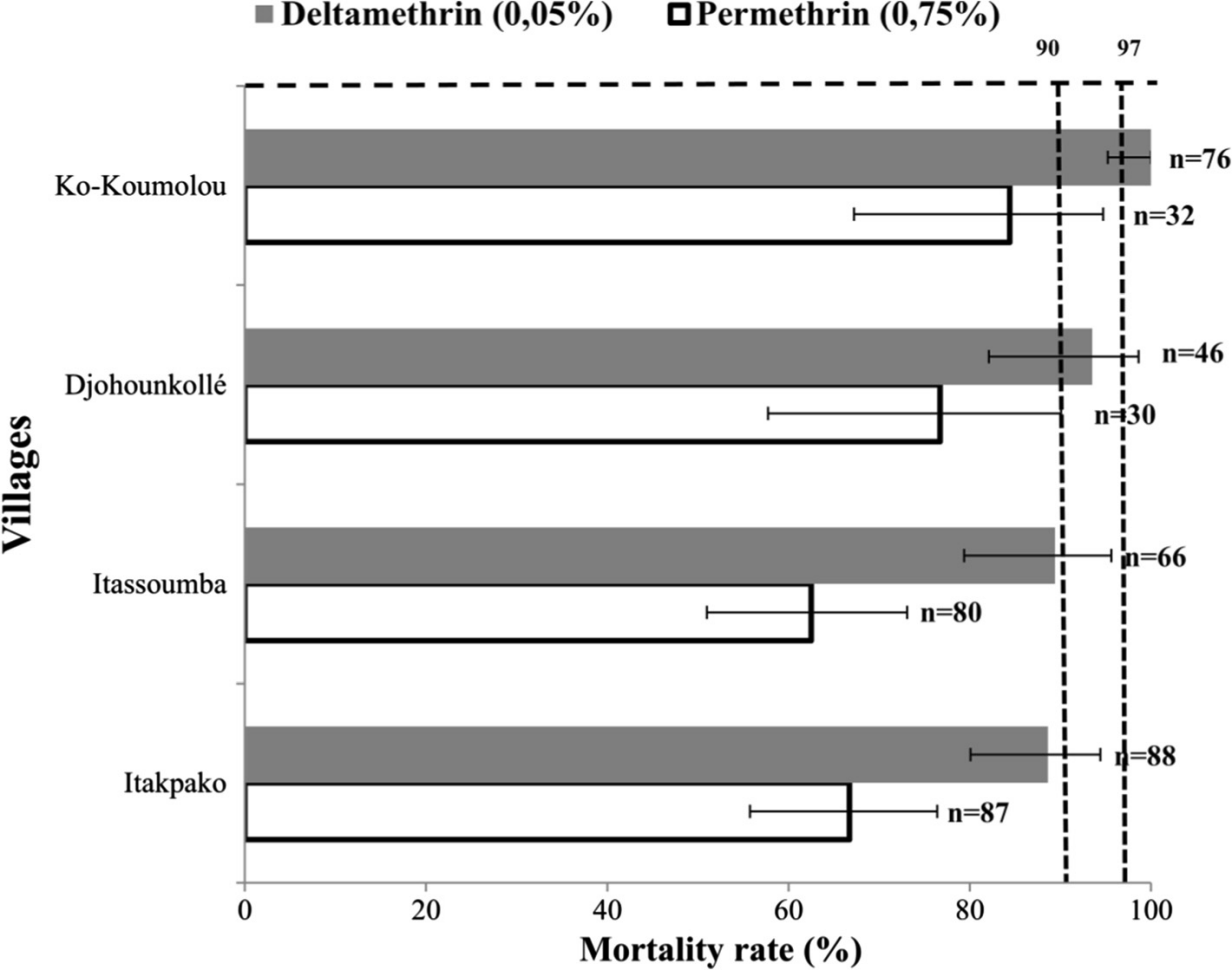
**Susceptibility level of *An. gambiae *** **populations to pyrethroids.**

The mortality rates in the control tubes were null. The induced mortality rates of the females of *An. gambiae* by permethrin was 66.67% (58/87), 62.50% (50/80), 76.67% (23/30) and 84.38% (27/32) respectively in Itakpako, Itassoumba, Djohounkollé and Ko-Koumolou (Figure 3). A strong resistance of the *An. gambiae* population to permethrin was thus observed in all the villages. No significant difference was observed between the mortality rates (p = 0.11).

On the other side, the resistance level to deltamethrin was not the same in all the villages. In Ko-Koumolou, the populations of *An. gambiae* were susceptible to deltamethrin [100% mortality rate (76/76)]. Suspected resistance was observed in Djohounkollé [93.48% mortality rate (43/46)]. In Itassoumba and Itakpako, the population of *An. gambiae* was resistant to deltamethrin with respective mortality rates of 89.39% (59/66) and 88.64% (78/88) (Figure 3).

As shown above, *An. gambiae* has developed a strong resistance to permethrin in the four villages. Deltamethrin showed a gradient of resistance levels. Considering the two insecticides tested, the populations of *An. gambiae* collected in Itassoumba and Itakpako seem more resistant.

### Frequencies of the *kdr* mutation in molecular forms and in dead and alive *An. gambiae* to deltamethrin

A total of 148 *Anopheles* resulting from the susceptibility tests were analyzed by PCR for the species, molecular forms and *kdr* mutation detection. In the four villages, the PCR revealed only the presence of *An. gambiae s.s.* with the two molecular M and S forms, but with the dominance of the M molecular form (M: 93.65%; S: 6.35%). The presence of the *kdr* mutation was also noted. This mutation was observed in the two molecular forms but at a variable frequency. The relative frequency of the forms is recorded in Table 4.

**Table 4:** **Genotypes and frequencies of *kdr *** **mutation in molecular forms and in dead and alive *An. gambiae *** **to deltamethrin**

**Villages**	**Total**	**Species**	**Molecular forms**		***kdr *** **Genotypes**			**F ( *kdr *)**	**Dead/Alive**	***kdr *** **Genotypes**			**F ( *kdr *)**	**p**
		**(Ag)**			**RR**	**RS**	**SS**			**RR**	**RS**	**SS**		
Itakpako	37	37	M	32	23	8	1	84.38	Dead	18	7	2	79.63^b^	0.74
			S	5	2	2	1	60						
			M + S	37	25	10	2	81.08^a^	Alive	7	3	0	85^b^	
Itassoumba	30	30	M	30	21	9	0	85	Dead	15	8	0	82.61^c^	0.43
			S	0	0	0	0	-						
			M + S	30	21	9	0	85^a^	Alive	6	1	0	92.86^c^	
Djohounkollé	43	43	M	37	22	15	0	79.73	Dead	20	14	0	79.41^d^	0.89
			S	0	0	0	0	-						
			M + S	37	22	15	0	79.73^a^	Alive	2	1	0	83.33^d^	
Ko-Koumolou	38	38	M	36	27	8	1	86.11	Dead	29	8	1	86.84	
			S	2	2	0	0	100						
			M + S	38	29	8	1	86.84^a^	Alive	-	-	-	-	

Ag: *An. gambiae*; ^a, b, c, d^*kdr* frequencies with the same superscript are statistically similar (p > 0.05).

As shown by the results, the *kdr* frequency is similar in dead and alive *An. gambiae* mosquitoes in Itakpako, Itassoumba and Djohounkollé (p > 0.05). Moreover, the frequency of the *kdr* mutation is high and similar in all the villages: 86.84% in Ko-Koumolou; 81.08% in Itakpako, 79.73% in Djohounkollé and 85% in Itassoumba (p = 0.63).

## Discussion

Our study revealed the presence of 13 mosquito species in the study areas. This result is similar to that of Huttel [27] who obtained 14 species in Southern Benin with the same sampling technique. In Djohounkollé, we collected a large number of *Aedes vittatus* compared to *Culex* and *Mansonia*. This strong presence of *Aedes vittatus* in this village surprised us because it is a mosquito that is mainly found in hilly areas; this is not the case of Djohounkollé. Indeed, these mosquitoes usually lay their eggs in standing water in the hollows of rocks [28]. However, after our investigations in the village, we noticed a strong presence of larvae and pupae of this mosquito in open and plaster tanks containing rainwater. This water is used for the traditional extraction of palm oil during the short dry season (period of harvest palm nuts). The similarity of these tanks with hollow rocks, may be why *Aedes vittatus* females prefer these tanks for oviposition.

The ecological characteristics of Itassoumba larval habitats resulting from the land use affect the density of anophelines. Indeed, the breeding sites resulting from the production of vegetables generally contain clean water favorable to the development of *Anopheles* larvae. Due to the nature of the soil, the water seeps quickly, but it is renewed when watering. The possibility that the pollution by accumulation of organic products is very low. Thus, the scarcity of polluted breeding sites is not conducive to the development of culicines larvae. In addition, fishponds also contain clean water regularly renewed to prevent their pollution by accumulation of organic matters that could be detrimental to fishes. This opportunity facilitates the development of anopheline larvae in particular *An. gambiae* to the detriment of culicine larvae in Itassoumba. This does not exist in the other three villages.

*An. gambiae s.s.* is the main vector involved in malaria transmission in the study localities. The vectorial role of *An. gambiae* in malaria transmission in Benin is reported in several works [4-15]. The predominance of the M molecular form of *An. gambiae* observed in the four villages is explained by the fact that, this form often finds favorable conditions for its development in Southern Benin. According to Coluzzi *et al.*[29], the M and S molecular forms of *An. gambiae* have variable distribution according to the ecological habitat and comprise five chromosomal forms in West Africa. The forms are Mopti, Savannah, Bissau, Forest and Bamako. Environmental analyzes showed that altitude, precipitation and temperature are the significant factors contributing in the spatial distribution of these forms [30]. Precipitation is a significant factor implicated in the development of the M and S molecular forms. The M molecular form of *An. gambiae* is described to be favorable to the wetlands where their breeding sites are permanent. This condition was the same in our study area.

The recorded aggressive densities of mosquitoes are significantly different (P < 0.05) between Itassoumba and the three other villages. We recorded higher mosquito density in Itassoumba during the twelve months of the study in the dry season and the rainy season, compared to the three other villages where the mosquito density was very low regardless of the season. A similar result was reported by Akogbéto [4] and Akogbéto *et al.*[31], which showed that malaria transmission depends on ecological features. The higher *An. gambiae s.s.* density observed in Itassoumba is justified by the presence and the maintenance of fishponds basins which constitutes breeding sites for the development of aquatic stages of mosquitoes during all year round. However, the predatory behavior of fishes in the breeding sites was not observed as expected. Instead *An. gambiae* larvae compete with the fishes for provender thrown on the surface of water. According to Protopopoff *et al.,*[32], the *Anopheles* density is higher in localities at the water edges than in those that are distant. The dynamics of *Anopheles* populations is in connection with the presence of the mosquito breeding sites and their productivity. Compared to Itassoumba, the low aggressive densities recorded throughout the year in Itakpako, Ko-Koumolou and Djohounkollé were due to low availability of breeding sites in these villages. Moreover, the soils in these villages are of a sandy-clay type allowing a fast infiltration of water, justifying therefore the temporary mosquito breeding sites there.

The infectivity rates of the *Anopheles* are similar in the study villages, except in Ko-Koumolou where the sporozoitic index is significantly higher. However, the number of *An. gambiae* analyzed by ELISA was very low: only 17 mosquitoes of which 5 were positive; that does not allow a valid conclusion. Despite the higher *Anopheles* density recorded in Itassoumba, the sporozoitic index was as high as that observed in Itakpako, Djohounkollé. As a result of this higher density, *An. gambiae* has maintained a closer contact with their host, which suggests the idea of precariousness and the decrease of the effectiveness of the distributed LLINs in Itassoumba. In Itassoumba, the relatively high sporozoitic index associated with a higher biting rate led to an extremely higher transmission of about 1159.62 infective bites of *An. gambiae* per man per year, roughly 3 infective bites per man per night. This rate was never recorded in Benin.

The spatial heterogeneity of the EIR reveals the importance of the local conditions on the intensity of malaria transmission which is an environmental problem [4].

Moreover, Yadouléton *et al.*[11] found an EIR of 168.18 infective bites/man/year in households close to market gardens of Houéyiho in southern Benin. There is a significant difference between this EIR and that of Itassoumba (1159.62 infective bites/man/year) (p < 0.0001). This result clearly shows that the combination of the fishponds with the market gardening has a significant impact on malaria parasite transmission.

A survey carried out in the market-gardening areas of Itassoumba, revealed that the farmers do not use the agricultural insecticides because of the intoxication risk of the fishes which are in the breeding basins. The other three villages are also characterized by a traditional agriculture practice which does not require the use of insecticides. The resistance observed in these localities could be explained by the use of the repellents and the LLINs in the households to fight mosquitoes. According to Protopopoff *et al.*[33], the selection of pyrethroids resistance in malaria vectors is due to the extensive use of LLINs. The same observation had been reported in Niger at the time of the implementation of a large scale national campaign distribution of LLINs to the whole population [34]. It is also possible that the emergence of resistance to pyrethroids in the four villages could be a consequence of several other factors (streaming of water with insecticide particles coming from the north of the country where pesticides are used massively against cotton pests etc.) other than the LLINs distributed across the country [35]. Under these conditions, the uncontrolled use of insecticides becomes a serious problem for public health. In permanent contact with insecticides, the populations of *An. gambiae* develop resistance toward these insecticides under several mechanisms. Thus, in our study, the *kdr* (Leu-Phe) mutation was investigated as the main mechanism involved in this resistance. This resistance is explained by the replacement of an amino acid by another, primarily the replacement of leucine by phenylalanine in West Africa (*kdr* Leu-Phe). This mutation was found in the two molecular forms M and S of *An. gambiae s.s*. This result is similar to that obtained by Dabiré et *al.*[36], which investigated the presence of the *kdr* mutation in the molecular forms M and S of *An. gambiae* in Bissau (capital of the Republic of Guinea Bissau). In addition, the similar *kdr* frequency recorded from dead and alive *An. gambiae* mosquitoes to deltamethrin in Itakpako, Itassoumba and Djohounkollé suggests the involvement of other resistance mechanisms to pyrethroids in addition to the *kdr.*

It is possible that an over expression of *CYP6M2* and *CYP6P3* genes, involved in the metabolism of the pyrethroids could also be implicated in this resistance. But our study did not explore this track. This phenomenon was carried out by Djouaka et *al*. [37] in the resistant populations of *An. gambiae* from Porto Novo (Capital of Benin).

## Conclusion

The present study has confirmed the heterogeneous character of malaria epidemiology. The four villages of the districts of Ifangni and Sakété, in spite of their proximity within a 15 kilometers radius, do not have the same level of transmission. The creation of fishponds and the regional market-gardening development in Itassoumba have caused a proliferation of *Anopheles*, especially *An. gambiae*. This proliferation is due to the presence of fishponds and water reserves (for watering vegetables), which also serves as breeding sites of *An. gambiae*. Owing to the permanent presence of these artificial *An. gambiae* breeding sites, the mosquito biting rates are very high in Itassoumba. In the villages located far from the agricultural development area, the *An. gambiae* breeding sites are formed only during the rainy season and the mosquito biting rates are very low with a ratio of 1 bite per man per year against 100 bites for the same period in Itassoumba. These results show that the land use can increase the level of malaria transmission. The case of Itassoumba is impressive: 9.62 to 21.65 infected bites of *An. gambiae* per human per year in Djohounkollé, Itakpako and Ko-Koumolou against 1159.62 in Itassoumba. It is also important to think about a strategy for managing resistance observed in the study villages because this could be a serious obstacle to the LLINs effectiveness.

Considering the very high level of transmission observed in Itassoumba, it is preferable that, the populations sleep under LLINs to avoid mosquito bites. We recommend that the human dwellings be located far from these agricultural activities in order to avoid the proximity of the populations to the mosquito breeding sites. It is also important to target the exact areas where high transmission is persisting such as Itassoumba so that the control operations can be more prioritized and focused in these areas.

## Competing interests

The authors declare that they have no competing interests.

## Authors’ contributions

AS, RG, FT, MO and MA conceived the study. AS, RG, MA and AM have participated in the design of the study. AS, ASS, HH, RO, RA and FA carried out the field activities and the laboratory analyses. VG has contributed to the mapping. AS, CA and MA drafted the manuscript. AS, RG, FT, RA, DG and AM critically revised the manuscript for intellectual content. All authors read and approved the final manuscript.

## References

[B1] Carnevale P Robert V Molez JF Baudon D Epidémiologie générale: faciès épidémiologiques des paludismes en Afrique subsaharienne Etudes médicales 1984 6 123 133

[B2] Mouchet J Carnevale P Coosemans M Ravaonjanahary C Richard A Robert V Typologie du paludisme en Afrique Cahiers Santé 1993 6 320 338

[B3] Fontenille D Lepers JP Campbell GH Coluzzi M Rakotoarivony I Coulanges P Malaria transmission and vector biology in Manarintsoa, high plateaux of Madagascar Am J Trop Med Hyg 1990 6 2 107 115 2202220 10.4269/ajtmh.1990.43.107

[B4] Akogbéto M Etude des aspects épidémiologiques du paludisme côtier lagunaire au Bénin 1992 Thèse de Doctorat ès-sciences: Université de Paris XI

[B5] Doannio JMC Dossou-Yovo J Diarrassouba S Rakotondraibe ME Chauvancy G Chandre F Riviere F Carnevale P La dynamique de la transmission du paludism à Kafiné, un village rizicole en zone de savane humide de Côte d’Ivoire Bull Soc Pathol Exot 2002 6 11 16 12012955

[B6] Koudou BG Tano Y Doumbia M Nsanzabana C Cissé G Girardin O Dao D N’goran EK Vounatsou P Bordmann G Keiser J Tanner M Utzinger J Malaria transmission dynamics in central Côte d’Ivoire: the influence of changing patterns of irrigated rice agriculture Med Vet Entomol 2005 6 27 37 10.1111/j.0269-283X.2005.00530.x 15752174

[B7] Meunier JY Safeukui I Fontenille D Boudin C Etude de la transmission du paludisme dans une future zone d’essai vaccinal en forêt équatoriale du sud Cameroun Bull Soc Pathol Exot 1999 6 309 312 10690465

[B8] Keiser J Castro M Maltese M Bos R Tanner M Effect of irrigation and large dams on the burden of malaria on a global and regional scale Am J Trop Med Hyg 2005 6 392 406 15827275

[B9] Manga L Bouchite B Toto JC Froment A La faune anophélienne et la transmission du paludisme dans une zone de transition forêt-savane au centre du Cameroun Entomol médicale 1997 6 4 9

[B10] Klinkenberg E McCall PJ Michael DW Amerasinghe FP Donnelly MJ Impact of urban agriculture on malaria vectors in Accra, Ghana Malar J 2008 6 151 10.1186/1475-2875-7-151 18680565 PMC2515328

[B11] Yadouléton A N’Guessan R Allagbé H Asidi A Boko M Osse R Padonou G Gazard K Akogbéto M The impact of the expansion of urban vegetable farming on malaria transmission in major cities of Benin Parasit Vectors 2010 6 118 10.1186/1756-3305-3-118 21143999 PMC3014909

[B12] Carnevale P Guillet P Robert V Fontenille D Doannio J Diversity of malaria in rice growing areas of Afrotropical region Parassitologia 1999 6 273 276 10697868

[B13] Yadouleton AW Asidi A Djouaka RF Braϊma J Agossou CD Akogbéto MC Development of vegetable farming: a cause of the emergence of insecticide resistance in population of *An. gambiae* in urban areas of Benin Malar J 2009 6 103 10.1186/1475-2875-8-103 19442297 PMC2686728

[B14] Akogbéto MC Djouaka R Noukpo H Use of agricultural insecticides in Benin Bull Soc Pathol Exot 2005 6 400 405 16425724

[B15] Padonou G Sezonlin M Gbedjissi G Ayi I Azondekon R Djenontin A Bio-Bangana S Oussou O Yadouleton A Boakye D Akogbeto M Biology of *anopheles gambiae* and insecticide resistance: entomological study for a large scale of indoor residual spraying in South East Benin J Parasitol Vector Biol 2011 6 59 68

[B16] Akogbeto MC Djouaka RF Kinde-Gazard DA Screening of pesticide residues in soil and water samples from agricultural settings Malar J 2006 6 22 10.1186/1475-2875-5-22 16563153 PMC1513586

[B17] Ministère de la Santé Annuaire des statistiques sanitaires 2011 2012 Cotonou: Direction de la Programmation et de la Prospective

[B18] Ministère de la Santé Annuaire des statistiques sanitaires 2012 2013 Cotonou: Direction de la Programmation et de la Prospective

[B19] Gillies M De Meillon B The *Anophelinae* of Africa south of the Sahara Publ South Afri Inst Med Res 1968 6 343

[B20] WHO Recommandations de la consultation technique sur la lute contre les vecteurs du paludisme dans la Région africaine de l’OMS 2011 Brazaville: Congo/ Rapport technique de l’OMS 2

[B21] Burkot TR Williams JL Schneider I Infectivity to mosquitoes *of Plasmodium falciparum* clones grown in vitro from the same isolate Trans R Soc Trop Med Hyg 1984 6 339 341 10.1016/0035-9203(84)90114-7 6380022

[B22] Lombardi Espositof Zavala F Lamizana L Rossip Saba-Tinelli G Nussenzweirg S Coluzzi M Detection and anatomical localisation of *Plasmodium falciparum* circumsporozoïte protein and sporozoïtes in the Afrotropica1 malaria vector *Anopheles gambiae s.1* Am J Trop Med Hyg 1987 6 491 494 3318517 10.4269/ajtmh.1987.37.491

[B23] Scott J Brogdon W Collins F Identification of single specimens of *Anopheles gambiae* complex by polymerase chain reaction Am J Trop Med Hyg 1993 6 520 529 8214283 10.4269/ajtmh.1993.49.520

[B24] Favia G Della Torre A Bagayoko M Lanfrancotti Sagnon NF Toure Y Coluzzi M Molecular identification of sympatric chromosomal forms of *Anopheles gambiae* and further evidence of their reproductive isolation Insect Mol Biol 1997 6 377 383 10.1046/j.1365-2583.1997.00189.x 9359579

[B25] Martinez-Torres D Chandre F Williamson M Darriet F Bergé J Devonshire A Guillet P Pasteur N Pauron D Molecular characterization of pyrethroid knockdown resistance (*kdr*) in the major malaria vector *Anopheles gambiae* s.s Insect Mol Biol 1998 6 179 184 10.1046/j.1365-2583.1998.72062.x 9535162

[B26] R Development Core Team A language and environment for statistical computing 2011 Vienna, Austria: R Foundation for Statistical Computing URL http://www.r-project.org/

[B27] Huttel J Note sur la répartition des moustiques dans le Bas-Dahomey Bull Soc Path Exot 1950 6 563 566

[B28] Irving-Bell RJ Inyang EN Tamu G Survival of Aedes vittatus (Diptera: Culicidae) eggs in hot, dry rockpools Trop Med Parasitol 1991 6 1 63 66 2052860

[B29] Coluzzi M Sabatini A Petrarca V Di Deco MA Chromosomal differentiation and adaptation to human environments in the *Anopheles gambiae* complex Trans R Soc Trop Med Hyg 1979 6 483 497 10.1016/0035-9203(79)90036-1 394408

[B30] De Souza D Kelly-Hope L Lawson B Wilson M Boakye D Environmental Factors Associated with the Distribution of *Anopheles gambiae* s.s in Ghana; an Important Vector of Lymphatic Filariasis and Malaria PLoS ONE 2010 6 9927 5 10.1371/journal.pone.0009927 PMC2847902 20360950

[B31] Akogbeto MC Padonou GG Bankole HS Gazard DK Gbedjissi GL Dramatic decrease in malaria transmission after large-scale indoor residual spraying with bendiocarb in Benin, an area of high resistance of *anopheles gambiae* to pyrethroids Am J Trop Med Hyg 2011 6 586 593 10.4269/ajtmh.2011.10-0668 21976555 PMC3183760

[B32] Protopopoff N Van Bortel W CSPeybroeck N Van Geertruyden JP Baza D D’Alessandro U Coosemans M Ranking malaria risk factors to guide malaria control efforts in African highlands PLoS ONE 2009 6 8022 10.1371/journal.pone.0008022 PMC2778131 19946627

[B33] Protopopoff N Verhaeghen K Van Bortel W Roelants P Marcotty T Baza D D’alessandro U Coosemans M A high increase in kdr in *Anopheles gambiae* is associated with an intensive vector control intervention in Burundi highlands Trop Med Int Health 2008 6 1479 1487 10.1111/j.1365-3156.2008.02164.x 18983277

[B34] Czeher C Labbo R Arzika I Duchemin JB Evidence of increasing Leu-Phe knockdown resistance mutation in *Anopheles gambiae* from Niger following a nationwide long-lasting insecticide-treated nets implementation Malar J 2008 6 189 10.1186/1475-2875-7-189 18817574 PMC2562389

[B35] Padonou G Sezonlin M Ossé R Aizoun N Oké-Agbo F Oussou O Gbédjissi G Akogbéto M impact of three years of large scale Indoor Residual Spraying (IRS) and Insecticide Treated Nets (ITNs) interventions on insecticide resistance in *Anopheles gambiae s.l*. in Benin Parasit Vectors 2012 6 72 10.1186/1756-3305-5-72 22490146 PMC3379941

[B36] Dabiré KR Diabaté A Agostinho F Alves F Manga L Faye O Baldet T Distribution of the members of Anopheles gambiae and pyrethroid knock-down resistance gene (*kdr*) in Guinea-Bissau, West Africa Bull Soc Path Exot 2008 6 2 119 123 18543705

[B37] Djouaka RF Bakare AA Coulibaly ON Akogbeto MC Ranson H Hemingway J Strode C Expression of the cytochrome P450s, CYP6P3 and CYP6M2 are significantly elevated in multiple pyrethroid resistant populations of *Anopheles gambiae s.s*. from Southern Benin and Nigeria BMC Genomics 2008 6 538 10.1186/1471-2164-9-538 19014539 PMC2588609

